# Comparative Transcriptome Analysis between the Cytoplasmic Male Sterile Line NJCMS1A and Its Maintainer NJCMS1B in Soybean (*Glycine max* (L.) Merr.)

**DOI:** 10.1371/journal.pone.0126771

**Published:** 2015-05-18

**Authors:** Jiajia Li, Shaohuai Han, Xianlong Ding, Tingting He, Jinying Dai, Shouping Yang, Junyi Gai

**Affiliations:** Soybean Research Institute, National Center for Soybean Improvement, MOA Key Laboratory of Biology and Genetic Improvement of Soybean (General), National Key Laboratory of Crop Genetics and Germplasm Enhancement, Nanjing Agricultural University, Nanjing, Jiangsu, People’s Republic of China; Chinese Academy of Sciences, CHINA

## Abstract

**Background:**

The utilization of soybean heterosis is probably one of the potential approaches in future yield breakthrough as was the situation in rice breeding in China. Cytoplasmic male sterility (CMS) plays an important role in the production of hybrid seeds. However, the molecular mechanism of CMS in soybean remains unclear.

**Results:**

The comparative transcriptome analysis between cytoplasmic male sterile line NJCMS1A and its near-isogenic maintainer NJCMS1B in soybean was conducted using Illumina sequencing technology. A total of 88,643 transcripts were produced in Illumina sequencing. Then 56,044 genes were obtained matching soybean reference genome. Three hundred and sixty five differentially expressed genes (DEGs) between NJCMS1A and NJCMS1B were screened by threshold, among which, 339 down-regulated and 26 up-regulated in NJCMS1A compared to in NJCMS1B. Gene Ontology (GO) annotation showed that 242 DEGs were annotated to 19 functional categories. Clusters of Orthologous Groups of proteins (COG) annotation showed that 265 DEGs were classified into 19 categories. Kyoto Encyclopedia of Genes and Genomes (KEGG) analysis showed that 46 DEGs were assigned to 33 metabolic pathways. According to functional and metabolic pathway analysis combined with reported literatures, the relations between some key DEGs and the male sterility of NJCMS1A were discussed. qRT-PCR analysis validated that the gene expression pattern in RNA-Seq was reliable. Finally, enzyme activity assay showed that energy supply was decreased in NJCMS1A compared to in NJCMS1B.

**Conclusions:**

We concluded that the male sterility of NJCMS1A might be related to the disturbed functions and metabolism pathways of some key DEGs, such as DEGs involved in carbohydrate and energy metabolism, transcription factors, regulation of pollen development, elimination of reactive oxygen species (ROS), cellular signal transduction, and programmed cell death (PCD) etc. Future research will focus on cloning and transgenic function validation of possible candidate genes associated with soybean CMS.

## Introduction

Soybean (*Glycine max* (L.) Merr.) is an important source of plant protein and oil. However, low yield is a key factor restricting its development. The utilization of soybean heterosis is probably one of the potential approaches in the future yield breakthrough as was the situation in rice breeding in China. Cytoplasmic male sterility (CMS) plays an important role in the production of hybrid seeds [1]. However, the molecular mechanism of CMS in soybean remains unclear.

The transcriptome is the complete set of transcripts in a cell at a specific developmental stage or physiological condition, which can provide information on gene expression and gene regulation [2]. Transcriptome sequencing (RNA-seq) is a recently developed high-performance and comprehensive method of transcriptome analysis [3, 4]. Transcriptome analysis using RNA-seq technology has allowed for the comparison and analysis of thousands of genes within one experiment [5].

Liu et al. [6] analyzed differentially expressed genes between chili pepper cytoplasmic male sterile line 121A and its near-isogenic line-restorer line 121C at the transcriptional level using Solexa/Illumina technology, and found a group of key genes and significant pathways associated with male sterility. Wei et al. [7] conducted transcriptome analysis of differentially expressed genes in the process of development in wild type and nuclear male sterile cotton anthers using digital gene expression profiles, and illustrated that many key genes involved in anther development showed the opposite gene expression patterns in GMS mutant anthers compared with that of wild type anthers at the same development stage. Yan et al. [8] conducted analysis of genome-wide and high-throughput transcriptome sequencing on young floral buds of *B*. *napus* CMS line Nsa and its novel restorer line NR1 using Solexa/Illumina techniques, and found a group of candidate genes associated with male sterility. An et al. [9] compared the genomic expression profiles of fertile and sterile young flower buds of *pol*-CMS in *B*.*napus* by RNA-Seq,and found some unigenes controlling anther development were dramatically down-regulated in sterile buds. However, there is no related report on CMS in soybean so far.

The soybean cytoplasmic male sterile line NJCMS1A was developed through consecutive backcross procedures with the cultivar N8855 as donor parent and N2899 (designated as NJCMS1B afterwards) as recurrent parent [10–12]. So NJCMS1A and NJCMS1B were a pair of near-isogenic lines and fit for the study on the molecular mechanism of CMS in soybean. In the present paper, we tried to find important differentially expressed genes and metabolism pathways might related to the soybean CMS through the comparative transcriptome analysis between the flower buds of NJCMS1A and those of NJCMS1B using the Illumina sequencing technology.

## Materials and Methods

### Plant Materials

The soybean cytoplasmic male-sterile line NJCMS1A was developed through consecutive backcross procedures with the cultivar N8855 as donor parent and N2899 (designated as NJCMS1B afterwards) as recurrent parent [10–12]. The genotypes of NJCMS1A and NJCMS1B were designated as S (rr) and N (rr), respectively. Both of NJCMS1A and NJCMS1B had similar nucleus genetic background, but with different cytoplasm, so they are a pair of near-isogenic lines of isonuclear alloplasmic type. NJCMS1A and NJCMS1B were planted in the summer of 2012 and 2014 at Jiangpu Experimental Station, National Center for Soybean Improvement, Nanjing Agricultural University, Nanjing, Jiangsu, China. The male-sterile plants were identified through three kinds of methods including the dehiscence of anthers, germination rate of pollens, and performance of plants at maturity. Cytological observation showed that the male abortion of NJCMS1A occurred mainly at the early binucleate pollen stage [13]. So during the javascript:void(0);flowering period, the flower buds in different sizes before abortion stage were collected and pooled from NJCMS1A and NJCMS1B plants respectively, then immediately frozen in liquid nitrogen and stored at -80 °C for further use. The samples collected in the summer of 2012 were used to RNA-seq and qRT-PCR experiments. The samples collected in the summer of 2014 were used to further qRT-PCR, enzyme activity assay and sugar content analysis.

### Total RNA Extraction, cDNA Library Construction and Illumina Deep Sequencing

Total RNA (5 μg) from the flower bud tissue (0.5–0.8 g) of NJCMS1A and NJCMS1B respectively was extracted using the TRIzol kit (Invitrogen, Carlsbad, CA, USA). An Ultra-micro spectrophotometer NanoDrop 2000 (Thermo Fisher Scientific, Waltham, MA, USA) was used to detect total RNA concentration and purity. Biological analyzer Agilent 2100 (Agilent, Santa Clara, CA, USA) was employed to detect the integrity of RNA. A Truseq RNA Sample Prep Kit (Illumina, SanDiego, CA, USA) was employed in mRNA purification and cDNA library construction according to the manufacturer’s instructions. The cDNA library was amplified by PCR enrichment, and was examined by 2% electrophoresis agarose gel to recover PCR fragments. TBS380 micro fluorescence (QuantiFluor ST/P, Promega, Madison, WI, USA) was used for the quantification of the cDNA library. Illumina sequencing was conducted on a Hiseq 2000 sequencer (Hiseq 2000 Truseq SBS Kit v3-HS (200 cycles), Illumina). These experiments were completed by Shanghai Majorbio Bio-pharm Biotechnology Co. (http://www.majorbio.com, Shanghai, China).

### Data Analysis of RNA-Seq

Software SeqPrep (https://github.com/jstjohn/SeqPrep) and Condetri_v2.0.pl (http://code.Google.Com/p/condetri/downloads/detail? Name=condetri_v2.0.pl) were used to filter noises for the original sequencing reads. Sequencing saturation and coverage in the two cDNA libraries were performed by the RSeQC-2.3.2 software [14].The sequencing adapter sequence, low-quality reads, higher N rate sequences, and too short sequences were removed. The remaining high-quality reads were submitted for mapping analysis against soybean reference genome (ftp://ftp.jgi-psf.org/pub/compgen/phytozome/v9.0/early_release/Gmax_275_Wm82.a2.v1/,version Glyma2.0) using Tophat (http://tophat.Cbcb.umd.edu/) [15], allowing two base mismatches. The mapped reads was then assembled with Cufflinks (http://cufflinks.cbcb.umd.edu/) [16].

### Differential Expression Analysis

The expression quantity of each gene (fragments per kilobase of exon model per million mapped fragments, FPKM) was estimated by Cuffdiff software [17]. “FDR (False Discovery Rate) ≤ 0.05 [18, 19] and |Log_2_FC (Fold Change)| ≥ 1” were used as the threshold for judging the significant of gene expression difference.

### Gene Ontology (GO) Annotation, COG Annotation, and KEGG Enrichment Pathway Analysis

Gene Ontology (GO, http://www.geneontology.org/) and functional enrichment analysis were conducted on all identified differentially expressed genes (DEGs) using the Goatools software [20] (https://github.com/tang haibao/goatools) (*P* ≤ 0.05). Functional classification of Clusters of Orthologous Groups of proteins (COG) was conducted on all identified DEGs using Blastx 2.2.24+ software in the STRING9.0 database (http://string-db.org/). Finally, metabolic pathway analysis was performed on all identified DEGs in the Kyoto Encyclopedia of Genes and Genomes (KEGG) database (http://www.genome.jp/kegg/genes.html) using Blastx/Blastp 2.2.24+ and KOBAS (http://kobas.cbi.pku.edu.cn/home.do) [21].

### Quantitative Real Time-PCR (qRT-PCR) Analysis

Quantitative real time-PCR (qRT-PCR) analysis was used to verify the RNA-Seq gene expression pattern. Total RNA was extracted using the TRIzol kit (Invitrogen, Carlsbad, CA, USA). Then, cDNA was synthesized by reverse transcription with DNA enzyme purified RNA samples using PrimeScript RT Reagent kits with gDNA Eraser (PrimeScript RT reagent Kit with gDNA Eraser, Takara, Dalian, China) following the manufacturer’s protocols. Gene-specific qRT-PCR primers were designed based on reference unigene sequences with Primer Premier 5.0 software (Premier Biosoft International, Palo Alto, CA, USA), gene-specific primers for qRT–PCR and genes annotation were listed in S5 Table. The mixed solution of qRT-PCR reaction (25 μl) contained SybrGreen qRT-PCR Master Mix (2×concentration, Ruian Biotechnologies, Shanghai, China) 12.5 μl, reverse and forward primers (10 μM) 0.5 μl, cDNA 2 μl and ddH_2_O 9.5 μl. qRT-PCR was performed in an ABI 7500 FAST Real-Time PCR System (Applied Biosystems, Foster City, CA, USA). PCR conditions were 2 min at 95°C, followed by 40 cycles of heating at 95°C for 10 s and annealing at 60°C for 40 s. The *β-actin* gene was used as the internal control. 2^-(△△Ct)^ algorithm was used to calculate the relative level of gene expression, NJCMS1B sample served as the control. The relative level of gene expression greater than 1 was regarded as up-regulated and less than 1 was regarded as down-regulated. All qRT-PCR reactions were performed with three biological replicates.

### Enzyme Activity Assay and Sugar Content Analysis

Total ATPase activity was measured at 636 nm by the UV-spectrophotometer (Philes, Nanjing, China, http://www.philes.cn/) using the ultramicro total ATPase assay kit (Jiancheng, Nanjing, China, http://mall.njjcbio.com). One unit of the total ATPase activity was defined as 1 μmol of inorganic phosphate (Pi) generated from ATP decomposed by ATPase in per hour per milligram tissue protein (μmol·Pi/mg·Protein/hour).

Sucrose phosphate synthase (SPS) activity was measured at 290 nm by the UV- spectrophotometer (Philes, Nanjing, China, http://www.philes.cn/) using the sucrose phosphate synthase assay kit (Jiancheng, Nanjing, China, http://mall.njjcbio.com). One unit of the SPS activity was defined as 1 μmol of sucrose generated by converting the substrate required enzyme content in per minute per milligram tissue protein under 37°C condition (U/mg·Protein).

Soluble sugars (glucose, fructose, sucrose) and starch content were measured at 340 nm by the UV-spectrophotometer (Philes, Nanjing, China, http://www.philes.cn/) using the glucose-fructose- sucrose assay kit and starch assay kit (BioSenTec, France, http://www.biosentec.fr/), respectively. The content of various sugars (g/L) was calculated based on the formulas according to the instructions in kits. All enzyme activity assay and sugar content analysis experiments were performed with three biological replicates.

## Results

### Transcriptome Sequencing and Sequence Alignment

In this study, the transcriptome sequencing analysis of flower buds of the cytoplasmic male sterile line NJCMS1A and its near-isogenic maintainer NJCMS1B in soybean was conducted using an Illumina Hiseq 2000 sequencer. The original image data obtained by sequencing base-calling were the original sequence reads. Each read in the Solexa paired-end (PE) sequencing was 101 bp in length. There were 112.10 million reads and a 11.32 Gb original data sets produced during sequencing. After the raw data were trimmed, 57,382,380 clean reads for NJCMS1A sample and 45,599,106 for NJCMS1B sample were obtained (Table 1). All clean reads were matched to the soybean reference genome by Tophat software, allowing two base mismatches [15, 22]. As a result, 53,376,483 mapped reads for NJCMS1A sample and 42,066,351 for NJCMS1B sample were obtained, with an average matching rate of 92.64% (Table 1).

**Table 1:** Number of reads sequenced and mapped to soybean genome.

Reads	NJCMS1A	NJCMS1B	Sum
**Raw bases (bp)**	6,271,412,392	5,050,932,230	11,322,344,622
**Raw reads**	62,093,194	50,009,232	112,102,426
**≥Q20 (%)**	94.23	92.90	94.10
**Clean bases (bp)**	5,630,986,966	4,433,806,292	10,064,793,258
**Clean reads**	57,382,380	45,599,106	102,981,486
**≥Q20 (%)**	98.79	98.63	98.81
**Mapped reads**	53,376,483	42,066,351	95,442,834
**Proportion (%)**	93.02	92.25	92.64

Note: Sequence length was 2×101 bp, length of each read was 101 bp using double end sequencing.

### Saturation and Coverage Analysis of Sequencing

**Fig 1:**
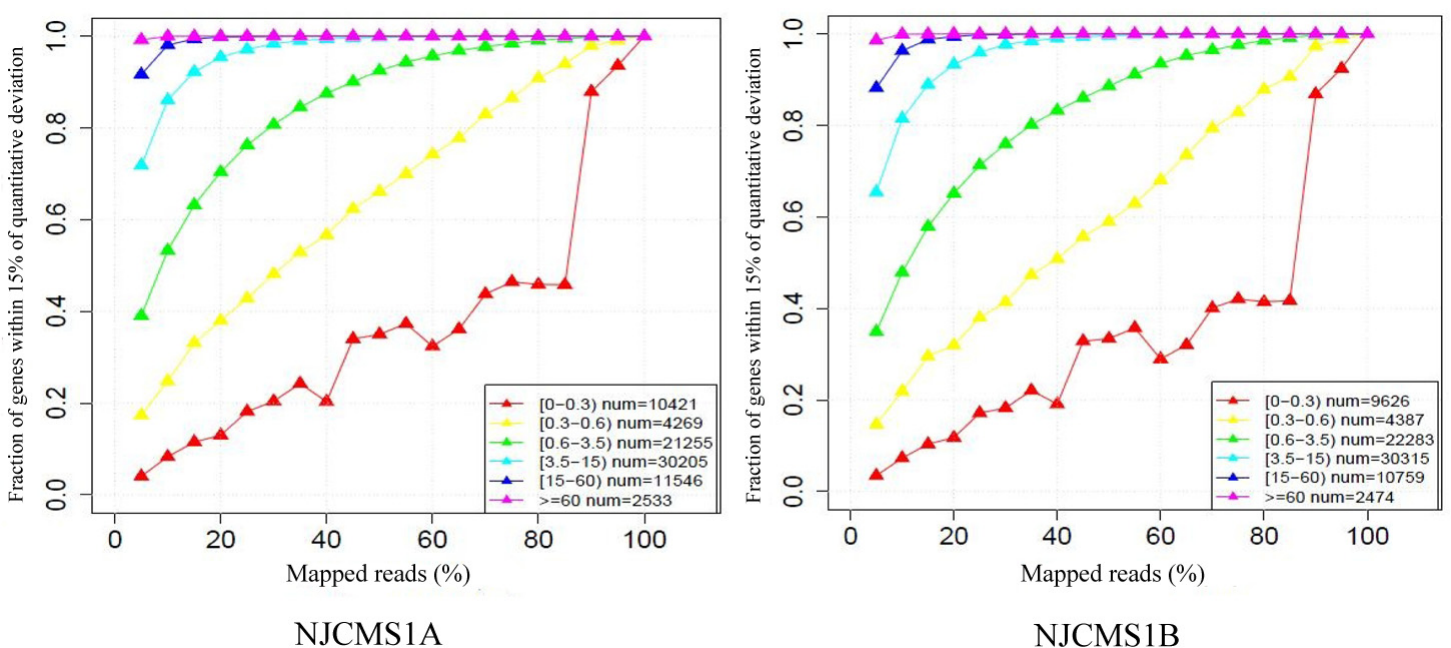
Saturation analysis of sequencing data of NJCMS1A and NJCMS1B. X-axis represented the percentage of mapped reads to soybean genome (%); Y-axis represented the fraction of genes within 15% of quantitative deviation. Each color line represented the saturation curve of different gene expression level, and the gene number within different FPKM interval was displayed in the lower right corner.

**Fig 2:**
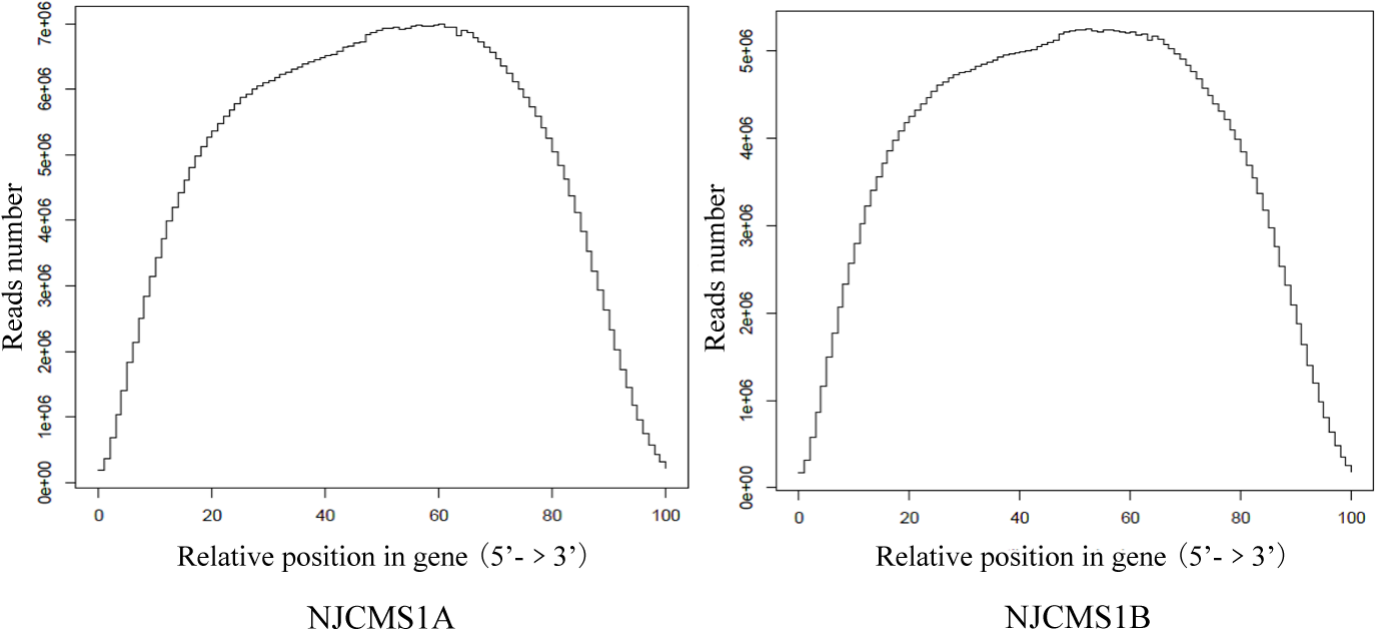
Coverage analysis of sequencing data of NJCMS1A and NJCMS1B. X-axis represented the relative position in gene, 0 and 100 indicated the 5’ and 3’ end of gene respectively; Y-axis represented the reads number mapped to soybean genome.

To estimate whether the sequencing depth was sufficient for the transcriptome coverage, the sequencing saturation and coverage in the two cDNA libraries were analyzed. Saturation analysis showed that most genes with moderate contents of expression (genes with greater than 3.5 FPKM) became saturated when more than 40% of sequencing reads were aligned (vertical axis numerical approached 1), which indicated that the overall quality of sequencing saturation in the two cDNA libraries was high, and sequencing amount covered the vast majority of expressed genes (Fig 1). Coverage analysis showed that two ends of the sequencing coverage in the two cDNA libraries had no significant peaks, which indicated that sequencing data among the two cDNA libraries was normally distributed (Fig 2).

### Analysis of Differentially Expressed Genes (DEGs)

A total of 88,643 transcripts were produced in the Illumina sequencing. Then 56,044 genes were obtained matching the soybean reference genome by Cufflinks software [16] (S1 Table). “FDR ≤ 0.05 and |Log_2_FC| ≥ 1” were used as the threshold to screen the DEGs between NJCMS1A and NJCMS1B. It was found that there were 365 DEGs between NJCMS1A and NJCMS1B (S2 Table), among which, 339 down-regulated and 26 up-regulated in NJCMS1A compared to in NJCMS1B. Furthermore, 93 down-regulated DEGs were only expressed in NJCMS1B and 9 up-regulated DEGs were uniquely expressed in NJCMS1A. Results showed that the number of the down-regulated DEGs was obviously larger than that of the up-regulated DEGs in NJCMS1A compared to in NJCMS1B. All of these RNA-Seq reads were deposited in Sequence Read Archive database (http://www.ncbi.nlm.nih.gov/Traces/sra/) under the Accession number SRP052011.

### Gene Ontology (GO) Annotation,COG Annotation and KEGG Enrichment Pathway Analysis

**Fig 3:**
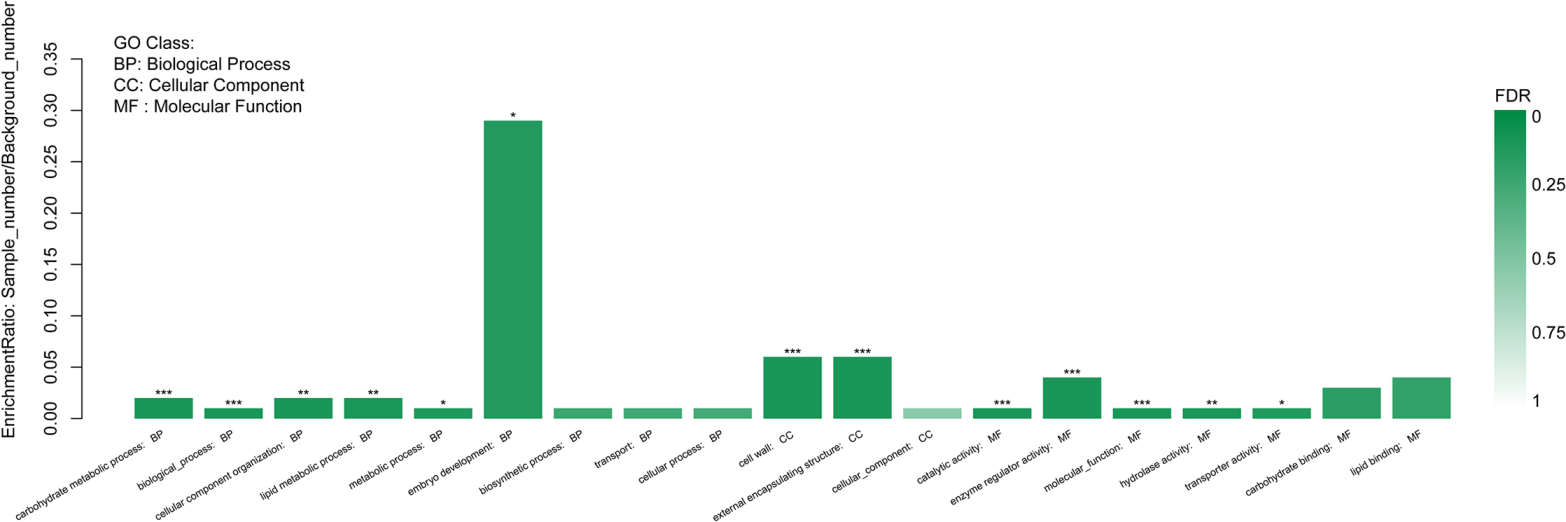
Gene Ontology functional analysis of differentially expressed genes between NJCMS1A and NJCMS1B. X-axis represented each GO term; Y-axis represented the enrichment ratio of genes in each main category.

Gene ontology (GO) is an internationally standardized gene function classification system used to describe properties of genes and their products in any organism, containing three ontologies: biological process, cellular component and molecular function [8]. In this study, plant GO Slim annotation was conducted by Blast2GO software (http://www.blast2go.com/b2ghome Version 2.3.5) [23]. Based on sequence homology, 242 DEGs were annotated to 19 functional categories, including 9 biological processes, 3 cellular components and 7 molecular functions (Fig 3, S3 Table). Among the biological process categories, “embryo development” was the main functional groups, followed by “cellular component organization” and “carbohydrate metabolic process”. Among the cellular component categories, “cellular component” was the main functional groups, followed by “external encapsulating structure”. Among the molecular function categories, “enzyme regulator activity” was the main functional groups, followed by “lipid binding” and “carbohydrate binding”.

**Fig 4:**
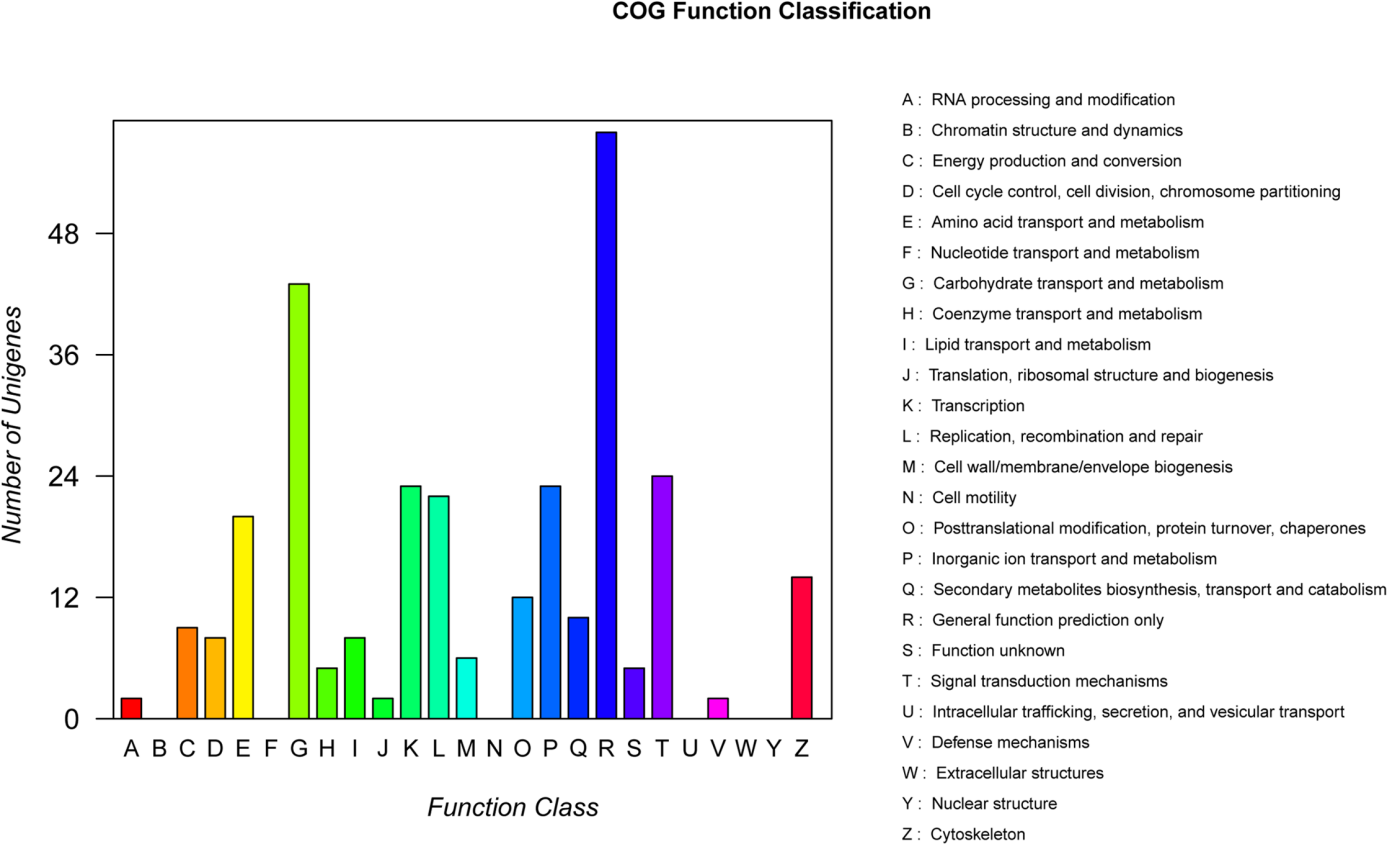
Function classification in Clusters of Orthologous Groups of proteins (COG) of differentially expressed genes between NJCMS1A and NJCMS1B. Capital letters on X-axis indicated the COG categories as listed on the right of the histogram; Y-axis indicated the number of differentially expressed genes.

All detected DEGs were blasted to STRING 9.0 for further annotation based on Cluster of Orthologous Groups (COG) protein categories [24]. A total of 265 DEGs were classified into 19 COG categories (Fig 4, S4 Table), among which, “general function prediction only” represented the largest group (58, 21.9%), followed by “carbohydrate transport and metabolism” (43, 16.2%), and "signal transduction mechanisms" (24, 9.1%). “RNA processing and modification” (2, 0.8%), “Translation, ribosomal structure and biogenesis” (2, 0.8%), and “Defense mechanisms” (2, 0.8%) were the smallest groups.

To identify the metabolic pathways in which the DEGs were involved and enriched, pathway-based analysis was performed using the KEGG pathway database [25]. In total, 46 DEGs were assigned to 33 KEGG pathways (Table 2), among which, “glycolysis/gluconeogenesis” was the most representative pathway (pathway: gmx00010, 8), followed by “carbon fixation in photosynthetic organisms” (pathway: gmx00710, 7), and “oxidative phosphorylation” (pathway: gmx00190, 6). Few DEGs were involved in “RNA transport” (pathway: gmx03013, 1) and “spliceosome” (pathway: gmx03040, 1) etc.

**Table 2:** KEGG pathways enriched of differentially expressed gene (DEGs) between NJCMS1A and NJCMS1B (core DEGs).

Gene Id	Gene Annotation	^a^Log_2_FC	^b^Regulation	^c^ *P* Value
***Glycolysis / Gluconeogenesis***				
Glyma.10G066700	Aldolase superfamily protein	-2.806566398	down	5.00E-05
Glyma.01G097400	Phosphoenolpyruvate carboxykinase 1	-2.759838312	down	5.00E-05
Glyma.11G247600	Glyceraldehyde-3-phosphate dehydrogenase C subunit 1	1.747097282	up	5.00E-05
Glyma.13G151800	Aldolase superfamily protein	-3.448031374	down	5.00E-05
Glyma.05G073100	Glyceraldehyde-3-phosphate dehydrogenase C2	-1.773605799	down	2.00E-04
Glyma.08G273100	Phosphoenolpyruvate carboxykinase 2	-3.29383008	down	5.00E-05
Glyma.01G118000	Thiamine pyrophosphate dependent pyruvate decarboxylase family protein	-4.408790632	down	5.00E-05
Glyma.19G078300	Glyceraldehyde-3-phosphate dehydrogenase C2	-2.910063151	down	5.00E-05
***Carbon fixation in photosynthetic organisms***				
Glyma.01G091000	Phosphoenolpyruvate carboxylase 4	-1.96777983	down	5.00E-05
Glyma.08G201200	NADP-malic enzyme 4	-3.040729085	down	5.00E-05
Glyma.02G130700	Phosphoenolpyruvate carboxylase 4	-1.854776055	down	5.00E-05
***Oxidative phosphorylation***				
Glyma.07G025900	H^(+)^-ATPase 9	-1.485862871	down	2.00E-04
Glyma.14G093300	NADH-ubiquinone oxidoreductase 20 kDa subunit, mitochondrial	-2.291938094	down	5.00E-05
Glyma.12G040300	Vacuolar H^+^-ATPase subunit E isoform 2	-2.813773122	down	5.00E-05
Glyma.07G048300	Pyrophosphorylase 1	-2.009730385	down	5.00E-05
Glyma.15G004300	H^(+)^-ATPase 9	-5.168319998	down	5.00E-05
Glyma.13G369300	H^(+)^-ATPase 9	-4.890112683	down	5.00E-05
***Cysteine and methionine metabolism***				
Glyma.17G184900	Cobalamin-independent synthase family protein	-4.042843442	down	5.00E-05
Glyma.05G090100	Cobalamin-independent synthase family protein	-2.797929869	down	5.00E-05
Glyma.11G254700	S-adenosyl-L-homocysteine hydrolase	-1.551923295	down	1.00E-04
Glyma.13G141600	Methionine adenosyltransferase 3	-2.981274586	down	5.00E-05
Glyma.10G054500	Methionine adenosyltransferase 3	-2.914574198	down	5.00E-05
***Starch and sucrose metabolism***				
Glyma.19G195400	Cell wall invertase 2	-2.346102727	down	5.00E-05
Glyma.08G308600	Sucrose phosphate synthase 3F	-1.784517228	down	1.00E-04
Glyma.03G223400	Plant invertase/pectin methylesterase inhibitor superfamily	-6.481838357	down	5.00E-05
Glyma.02G008300	VANGUARD 1 homolog 2	-3.847662291	down	5.00E-05
***Pentose and glucuronate interconversions***				
Glyma.19G020200	Pectate lyase family protein	-5.637503879	down	5.00E-05
Glyma.08G296900	Pectate lyase family protein	-5.903267116	down	5.00E-05
***Nitrogen metabolism***				
Glyma.02G035000	Carbonic anhydrase 2	-1.358114642	down	2.00E-04
Glyma.17G148000	Glutamate dehydrogenase 2	-1.532668021	down	2.00E-04
***Alanine*, *aspartate and glutamate metabolism***				
Glyma.02G241400	Glutamate decarboxylase	-2.026581623	down	5.00E-05
Glyma.14G211100	Glutamate decarboxylase	-3.278453751	down	5.00E-05
***Phagosome***				
Glyma.05G126100	Tubulin beta-7 chain	-1.482128236	down	2.00E-04
Glyma.01G197500	Tubulin alpha-2 chain	-1.512186909	down	5.00E-05
***Sphingolipid metabolism***				
Glyma.15G072800	Fatty acid desaturase family protein	-3.078035515	down	5.00E-05
***Stilbenoid*, *diarylheptanoid and gingerol biosynthesis***				
Glyma.08G089500	Cytochrome P450, family 81, subfamily D, polypeptide 3	0.532775756	up	5.00E-05
***Biosynthesis of unsaturated fatty acids***				
Glyma.14G121400	Plant stearoyl-acyl-carrier-protein desaturase family protein	-2.613215404	down	5.00E-05
***Ascorbate and aldarate metabolism***				
Glyma.07G225400	SKU5 similar 12	-3.417857252	down	5.00E-05
***Glutathione metabolism***				
Glyma.02G154400	Glutathione S-transferase 7	-3.023522601	down	5.00E-05
***Endocytosis***				
Glyma.18G289600	Heat shock cognate protein 70–1	-2.185713321	down	5.00E-05
***Phenylalanine metabolism***				
Glyma.08G292100	Peroxidase superfamily protein	-1.744501273	down	5.00E-05
***RNA transport***				
Glyma.10G212900	GTP binding Elongation factor Tu family protein	1.750839666	up	5.00E-05

^a^Log_2_FC ≥ 1 represented up-regulated and Log_2_FC ≤ -1 represented down-regulated.

^**b**^Regulation direction of DEGs (NJCMS1B was the control).

^**c**^ *P* value of ≤ 0.05 was considered statistically significant.

### Analysis of DEGs Potentially Related to Male-Sterility in Soybean

Carbohydrate and energy metabolism is one of the most basic metabolic pathways in biological metabolism. Its main physiological function is to provide required energy and carbon sources [26]. In this study, many DEGs were found to involve in carbohydrate and energy metabolism (Table 2), for example, there were 8 DEGs participated in glycolysis/gluconeogenesis pathway, among which, 7 DEGs were down-regulated and 1 DEG was up-regulated in NJCMS1A compared to in NJCMS1B; there were 7 DEGs participated in Carbon fixation in photosynthetic organisms and all down-regulated in NJCMS1A compared to in NJCMS1B; there were 4 DEGs participated in starch and sucrose metabolism and all down-regulated in NJCMS1A compared to in NJCMS1B. Three DEGs encoding H^(+)^-ATPase 9, one NADH-ubiquinone oxidoreductase 20 kDa subunit, one vacuolar H^+^-ATPase subunit and one pyrophosphorylase involved in the oxidative phosphorylation and all down-regulated in NJCMS1A compared to in NJCMS1B, and so on (See detail to Table 2).

Transcription factors are essential for the regulation of gene expression. Changes in gene transcription are associated with changes in expression of transcription factors [27]. Our results showed that there were 15 DEGs encoding transcription factors (S2 Table), among which, 14 DEGs were down-regulated and 1 DEG was up-regulated in NJCMS1A compared to in NJCMS1B. The 14 down-regulated DEGs were 7 zinc-finger family proteins, 2 WRKY family transcription factors, 1 phytochrome interacting factor 3-like 5, 1 sequence-specific DNA binding transcription factor, 1 MYB domain protein 101, 1 Ras-related small GTP-binding family protein, 1 F-box/RNI-like superfamily protein. One up-regulated DEG was F-box family protein with a domain of unknown function.

In the present study, 38 DEGs were found involved in regulation of pollen development (S2 Table). There were 34 DEGs participated in the pollen wall development and all down-regulated in NJCMS1A compared to in NJCMS1B, among which, 22 DEGs were might related to cell wall remodeling, including 13 genes encoding plant invertase/pectin methylesterase inhibitor superfamily proteins, 3 genes encoding pectin lyase-like superfamily proteins, 2 genes encoding pectate lyase family proteins, 1 gene encoding cell wall invertase 2, 1 gene encoding cellulose synthase like D4, 1 gene encoding callose synthase 5, 1 gene encoding hexokinase-like 3; the other 12 DEGs were might related to cytoskeletal structures, including 3 genes encoding myosin family proteins, 3 genes encoding profiling, 2 genes encoding tubulin, 2 genes encoding actin-11, 1 gene encoding myosin 2, and 1 gene encoding actin depolymerizing factor. In addition, there were 4 DEGs encoding pollen Ole e 1 allergen and extensin family proteins and all down-regulated in NJCMS1A compared to in NJCMS1B.

In this study, we also found other DEGs potentially related to male-sterility in soybean. There were 17 DEGs related to elimination of reactive oxygen species (ROS) (S2 Table), among which, 15 DEGs were down-regulated and 2 DEGs were up-regulated in NJCMS1A compared to in NJCMS1B. Fifteen down-regulated DEGs were 13 genes encoding late embryogenesis abundant protein (LEA) family proteins and 2 genes encoding peroxidase superfamily proteins. Two up-regulated DEGs were 1 gene encoding LEA family protein and 1 gene encoding alternative oxidase. In addition, 12 DEGs associated with calmodulin-like were found and all down-regulated in NJCMS1A compared to in NJCMS1B (S2 Table). They were 3 genes encoding calcium-binding EF-hand family proteins, 4 genes encoding calcium-dependent lipid-binding (CaLB domain) family proteins and 5 genes encoding calcium-dependent protein kinases. Notably, several DEGs with known or unknown function were also found in our results (S2 Table).

### Analysis of DEGs by qRT-PCR

**Fig 5:**
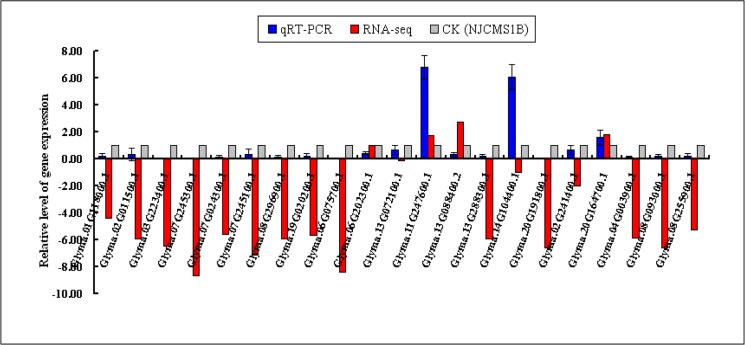
DEGs confirmed by qRT-PCR using the same sample as that in RNA-seq. X-axis represented gene name, the blue column represented qRT-PCR results, the red column represented RNA-seq results, and gray column represented CK (NJCMS1B); Y-axis represented the relative level of gene expression. Gene-specific qRT-PCR primers and gene name were listed in S5-1 Table. All qRT-PCR reactions were performed with three biological replicates.

**Fig 6:**
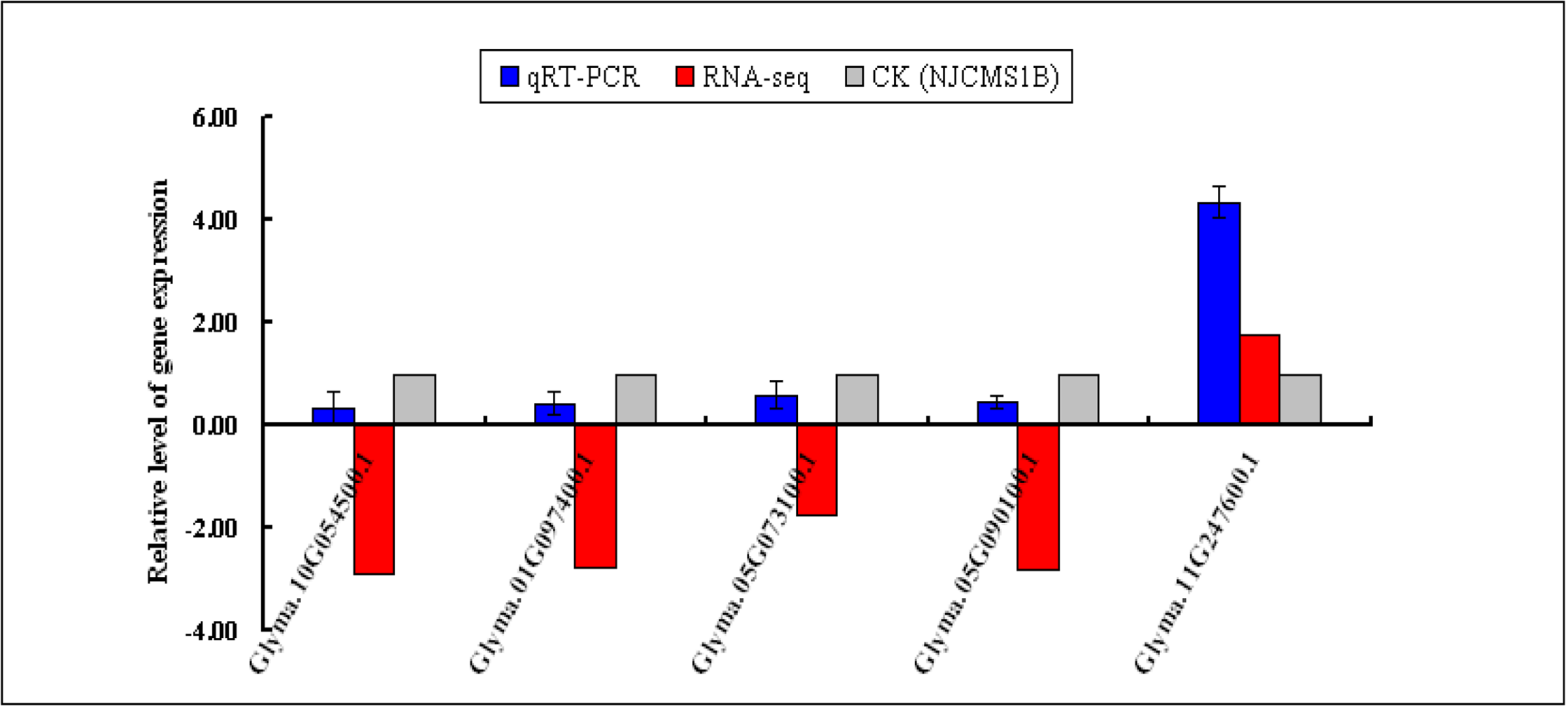
DEGs confirmed by qRT-PCR using different sample from that in RNA-seq. X-axis represented gene name, the blue column represented qRT-PCR results, the red column represented RNA-seq results, and gray column represented CK (NJCMS1B); Y-axis represented the relative level of gene expression. Gene-specific qRT-PCR primers and gene name were listed in S5-2 Table. All qRT-PCR reactions were performed with three biological replicates.

According to the functional and metabolic pathway analysis combined with previously reported literatures, 21 DEGs might related to CMS were chosen to be conducted qRT-PCR analysis using the same sample as that in RNA-seq. It was found that the expression patterns of qRT-PCR of 18 DEGs were consistent with those of RNA-Seq, while the other 3 DEGs were not (Fig 5, S5-1 Table). The coincidence rate between qRT-PCR results and RNA-Seq results was 85.71%. In addition, 5 DEGs might related to CMS were chosen to be conducted qRT-PCR analysis using different sample from that in RNA-seq (Fig 6, S5-2 Table). The results showed that the expression patterns of qRT-PCR of 5 DEGs were consistent with those of RNA-Seq. These results indicated that the RNA-Seq results in the present study were reliable.

### Enzyme Activity Assay and Sugar Content Analysis

**Fig 7:**
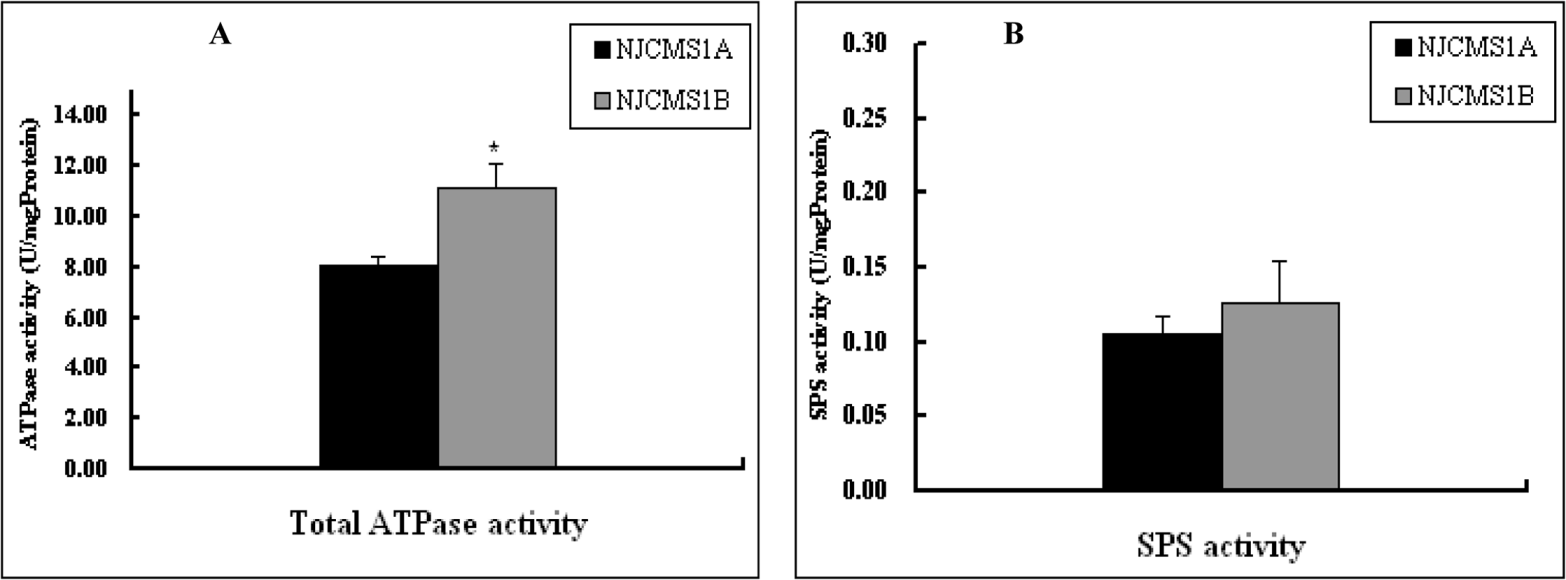
Enzyme activity assay in NJCMS1A and NJCMS1B. The black column represented NJCMS1A and gray column represented NJCMS1B on X-axis; Y-axis represented the enzyme activity. (A) Total ATPase activity and (B) Sucrose phosphate synthase (SPS) activity. The data were given as Mean ± SD from three biological replicates.

The ATPase, a key enzyme for the synthesis of ATP in cellular biosynthesis, plays an important role in material transport, energy transformation and information transmission [28]. Sucrose phosphate synthase (SPS) plays a key role for sucrose synthesis in photosynthetic organs of green plants. To investigate the enzymes activity associated with energy metabolism, we tested the total ATPase activity and SPS activity in flower buds of NJCMS1A and NJCMS1B. The results showed that the total ATPase activity was significantly decreased in male-sterile line NJCMS1A, relative to maintainer NJCMS1B (Fig 7), but, there was not significant difference of SPS activity between NJCMS1A and NJCMS1B (Fig 7), this might be caused by sample tissues and needed to be further studied.

**Fig 8:**
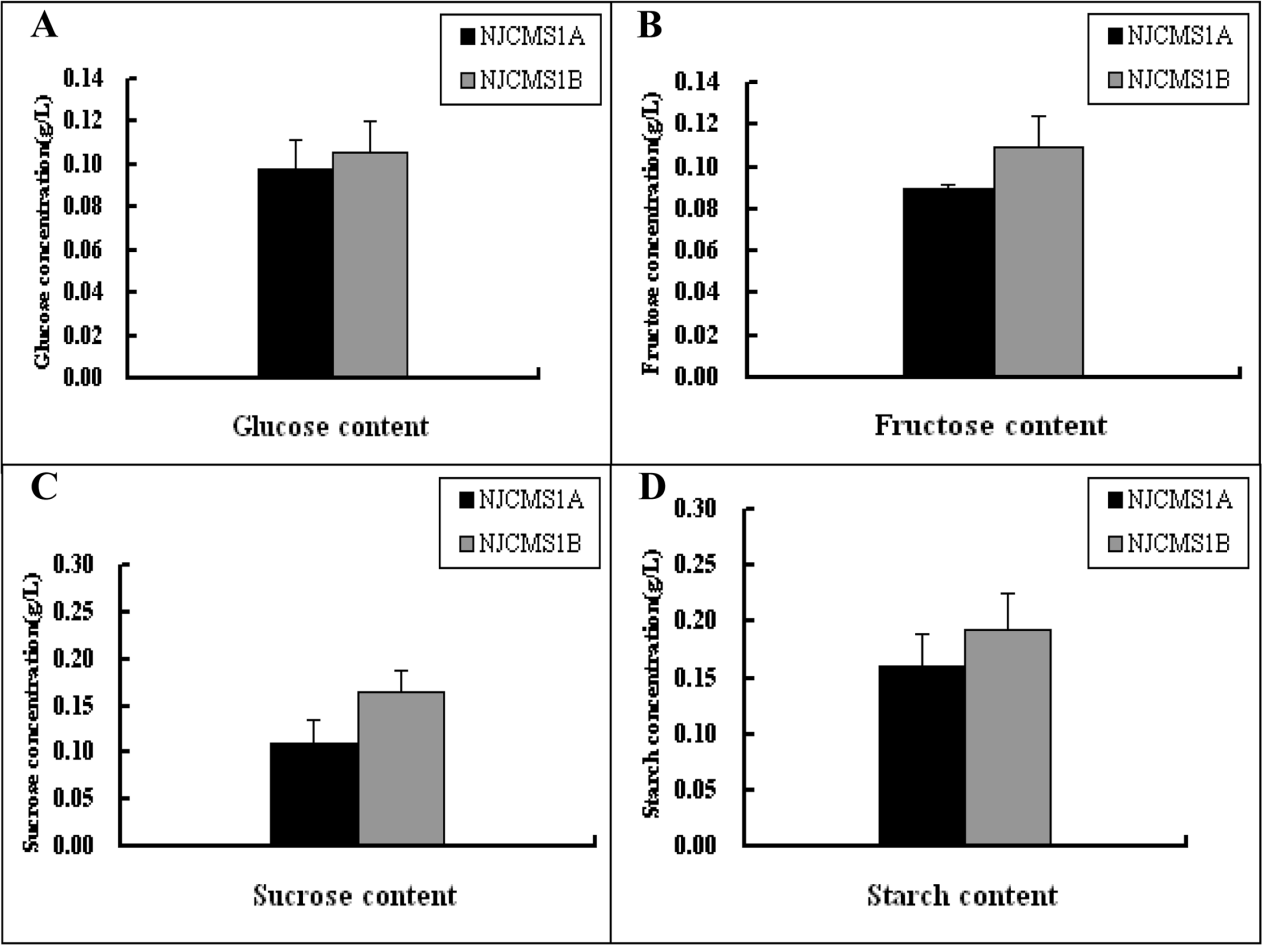
Sugar content analysis in NJCMS1A and NJCMS1B. The black column represented NJCMS1A and gray column represented NJCMS1B on X-axis; Y-axis represented sugar concentration. (A) Glucose; (B) Fructose; (C) Sucrose and (D) Starch. The data were given as Mean ± SD from three biological replicates.

To improve our understanding of the basis of energy deficiency in the male-sterile line, we tested the content of sugars, including glucose, fructose, sucrose and starch. The results showed that there were not significant difference of soluble sugars (glucose, fructose, sucrose) and starch content between NJCMS1A and NJCMS1B (Fig 8), this might be caused by sample tissues and needed to be further studied.

## Discussion

In the present study, the comparative transcriptome analysis between the cytoplasmic male sterile line NJCMS1A and its near-isogenic maintainer NJCMS1B in soybean was conducted using the Illumina sequencing technology. Three hundred and sixty five DEGs were screened between NJCMS1A and NJCMS1B by threshold, among which, 339 down-regulated and 26 up-regulated in NJCMS1A compared to in NJCMS1B. According to GO, COG and KEGG functional and metabolic pathway analysis combined with previously reported literatures, the relations between some key DEGs and the male sterility of NJCMS1A would be discussed as follows.

### Analysis of DEGs Involved in Carbohydrate and Energy Metabolism Potentially Related to CMS in Soybeans

The development of stamen and pollen in flowering plants is a complicated process that involves a series of well coordinated cytoplasmic and nuclear gene interactions leading to multifarious metabolic processes and structural changes [29]. Dorion et al. [30] stated that a dysfunction in a major metabolic pathway, such as sugar metabolism, could adversely affect the development of pollen grain. It has been suggested that a high respiration rate and great energy demand are usually observed during pollen development [31]. Carbohydrate not only provides nutrition for anther development, but also affects anther and pollen development as a signal substance [26]. Moreover, Bergman et al. [32] and Teixeira et al. [33] had proved that the ATP content related to supply of energy in male sterile lines was significantly decreased. In this study, many DEGs were found involved in carbohydrate and energy metabolism pathway (Table 2), and were down-regulated in the male sterile line NJCMS1A compared to in the near-isogenic maintainer NJCMS1B. Meanwhile, enzyme activity assay showed total ATPase activity was significantly decreased in NJCMS1A, relative to NJCMS1B. These results showed that the expression of genes related to the supply of energy in the male sterile line NJCMS1A was suppressed, which might result in a shortage in energy required for pollen maturation and the abnormal development of pollen, and ultimately led to the male sterility of NJCMS1A.

### Analysis of DEGs Encoding Transcription Factors Potentially Related to CMS in Soybeans

Hao et al. [27] indicated that a single transcription factor could regulate the expressions of multiple genes in a metabolic pathway. Yan et al. [8] determined that transcription factors were essential for the regulation of plant gene expression, and changes in gene transcription were related to changes in the expression of transcription factors. Alteration in the expression of transcription factor genes normally results in dramatic changes during plant growth [34, 35]. In this study, 14 and 1 DEG encoding transcription factors were found down-regulated and up-regulated in the male sterile line NJCMS1A compared to in the near-isogenic maintainer NJCMS1B respectively. This might lead to the expression of genes related to the development of flower organ in NJCMS1A was interfered, possibly resulted in male sterility of NJCMS1A.

### Analysis of DEGs Involved in Regulation of Pollen Development Potentially Related to CMS in Soybeans

Pollen development is an essential process of sexual reproduction in flowering plants. Zhu et al. [36] stated that pollen cell wall development in pollen grains ensured plant sexual reproduction, and the majority of male sterile traits were associated with abnormal wall development. Zhang et al. [37] determined that *BoPMEI1* expression was suppressed and resulted in the retardation of pollen development and partial male sterility in the antisense expression studies of *BoPMEI1* in *Arabidopsis thaliana*. Hideaki et al. [38] showed that genes involved in the cytoskeleton category played key roles in cell wall expansion. Li et al. [39] determined that the over-expression of actin depolymerizing factor (*GhADF7*) in *Gossypium hirsutum* L. could alter the balance of actin depolymerization and polymerization, resulting in incomplete cytokinesis and partial male sterility. In addition, recent studies have suggested that the pollen Ole e 1 allergen and extensin family protein functioned as developmental regulators in many plant tissues [40–42]. In this study, 38 DEGs were found related to the pollen development and all down-regulated in the male sterile line NJCMS1A compared to in the near-isogenic maintainer NJCMS1B, among which, 34 DEGs participated in the pollen wall development and 4 DEGs functioned as developmental regulators in different plant tissues. The above results showed that the abnormal expression of genes involved in the pollen development in NJCMS1A might directly influence the pollen development of NJCMS1A including cell wall remodeling, aberrant cytoskeletal structures, and lose of functions as developmental regulators in pollen etc., and ultimately resulted in the male sterility of NJCMS1A.

### Analysis of DEGs Involved in Elimination of ROS and Cellular Signal Transduction Potentially Related to CMS in Soybeans

It has been suggested that an abnormality of activated oxygen metabolism in the development of the anther or young panicle might be related to male sterility [43–45]. Li et al. [43] showed that a higher concentration of ROS and mitochondrial damage existed in the microspores of male sterile rice lines. Liu et al. [45] demonstrated that an important characteristic of LEA, which differed from that of other molecular chaperone protein functions, was that they could eliminate active oxygen and protect cell membrane stability. In the present study, 15 DEGs related to elimination of ROS were found down-regulated in the male sterile line NJCMS1A compared to in the near-isogenic maintainer NJCMS1B. The results showed that the expression of a variety of active oxygen scavenging enzyme genes was inhibited in NJCMS1A, resulting in higher concentrations of ROS in NJCMS1A than in NJCMS1B, which might be a possible reason for the occurrence of male sterility of NJCMS1A.

Several studies had demonstrated that, Ca^2+^, a messenger in cellular signal transduction, functioned as a pivotal regulator of the cell life cycle including cell division, differentiation, and apoptosis [46–49]. Rato et al. [50] showed that pollen development depended on multiple signaling pathways, in which calmodulin was a key element. In this study, 12 DEGs associated with calmodulin-like were identified and all down-regulated in the male sterile line NJCMS1A compared to in the near-isogenic maintainer NJCMS1B.The results indicated that their differential expression might cause destruction of calcium signaling pathways and abnormal pollen development, resulting in male sterility of NJCMS1A.

### Analysis of Other DEGs Potentially Related to CMS in Soybeans

It was suggested that aspartic protease acted as an anti-cell-death factor participating in programmed cell death (PCD) and the over-expression of this gene resulted in male sterility in *Arabidopsis* [51]. In this study, we found 1 gene encoding aspartic proteinase A1 up-regulated in NJCMS1A, the higher expression level of this gene in the male sterile line NJCMS1A could lead to PCD of the pollen cell, ultimately causing male sterility. In addition, other DEGs were also found in our study, for example, there were 2 genes encoding cytochrome P450 family protein, 1 gene encoding glutathione S-transferase tau 9, 1 gene encoding leucine-rich repeat transmembrane protein kinase and 1 gene encoding glyceraldehyde-3-phosphate dehydrogenase C subunit 1, etc., which were up-regulated in the male sterile line NJCMS1A compared to in the near-isogenic maintainer NJCMS1B; seven genes encoding major facilitator superfamily proteins, 6 genes encoding NAD(P)-binding Rossmann-fold superfamily proteins and 3 genes encoding tetratricopeptide repeat (TPR)-containing proteins, etc., which were down-regulated in the male sterile line NJCMS1A compared to in the near-isogenic maintainer NJCMS1B; and 20 DEGs with unknown functions. The above DEGs might be associated with the male sterility of NJCMS1A, but their specific functions needed to be further studied.

## Conclusions

In the present study, the comparative transcriptome analysis between the cytoplasmic male sterile line NJCMS1A and its near-isogenic maintainer NJCMS1B in soybean was conducted. The results showed that there were 365 DEGs between NJCMS1A and NJCMS1B, among which, 339 down-regulated and 26 up-regulated in NJCMS1A compared to in NJCMS1B. According to GO, COG and KEGG functional and metabolic pathway analysis combined with the previously reported literatures, we concluded that the male sterility of NJCMS1A might be related to the disturbed functions and metabolism pathways of some key DEGs, such as DEGs involved in carbohydrate and energy metabolism, encoding transcription factors, regulation of pollen development, elimination of ROS, cellular signal transduction, and PCD etc. These results will help to elucidate the molecular mechanism of CMS in soybean, and provides a theoretical basis for better utilization of soybean heterosis. Future research will focus on the cloning and transgenic function validation of possible candidate genes associated with soybean CMS.

## Supporting Information

S1 Table Total number of identified genes between NJCMS1A and NJCMS1B. (XLS)

S2 Table Number of differentially expressed genes between NJCMS1A and NJCMS1B. (XLS)

S3 Table Gene Ontology functional annontation of differentially expressed genes between NJCMS1A and NJCMS1B. (XLS)

S4 Table Clusters of Orthologous Groups of proteins classification of differentially expressed genes between NJCMS1A and NJCMS1B. (XLS)

S5 Table Comparison of expression patterns between RNA-seq and qRT-PCR. S5-1 Table. DEGs confirmed by qRT-PCR using the same sample as that in RNA-seq. S5-2 Table. DEGs confirmed by qRT-PCR using different sample from that in RNA-seq.
(XLS)
